# Insights into self-evaluated stress, anxiety, and depression among dental students

**DOI:** 10.1038/s41598-024-79427-7

**Published:** 2024-12-05

**Authors:** Doina Iulia Rotaru, Radu Marcel Chisnoiu, Sorana D. Bolboacă, Edith Ana Gileru, Andrea Maria Chisnoiu, Ada Gabriela Delean

**Affiliations:** 1 Department of Odontology, Endodontics and Oral Pathology, ″Iuliu Hațieganu″ University of Medicine and Pharmacy, 33 Moților Street, Cluj-Napoca, 400001 Romania; 2 Department of Medical Informatics and Biostatistics, ″Iuliu Hațieganu″ University of Medicine and Pharmacy, 6 Pasteur Street, Cluj-Napoca, 400349 Romania; 3 Individual psychology office, 423A Avram Iancu Street, Florești, 407280 Romania; 4 Department of Prosthodontics, ″Iuliu Hațieganu″ University of Medicine and Pharmacy, 32 Clinicilor Street, Cluj-Napoca, 400006 Romania

**Keywords:** Dental medicine, Depression, Anxiety, Stress, DASS-21, Human behaviour, Medical research

## Abstract

Stress, anxiety, and depression have a negative impact on students’ learning and academic performances. In our longitudinal study conducted on dental students from Cluj-Napoca and Oradea, the most representative university centers in north-west of Romania, we evaluated the prevalence and effect of exams on stress, anxiety, and depression, in association with socio-demographic factors. The students self-administered the DASS-21R questionnaire at the beginning of the academic year 2022–2023, before the Christmas holiday, during the winter session, and after the winter session. Students from Cluj-Napoca had higher stress scores at the beginning of the academic year compared to those from Oradea (6.8% vs. 3.7%, P-value < 0.05), especially the females and students with no previous university graduations. Anxiety and depression were significantly more frequently reported by students who study in Cluj-Napoca compared to those who study in Oradea (anxiety 37% vs. 28.2%, P-value < 0.05; depression 17% vs. 11%, P-value < 0.05). We did not observe any significant differences in DASS-21R scores regarding summer jobs, foreign language knowledge, or monthly income. Our results support the needs for interventions regarding stress, anxiety, and depression implemented from the very beginning of university studies and, along with strategies for stress prevention and management of these conditions.

## Introduction

The Richard S. Lazarus Theory of Stress (1966), developed by Cohen (1977) and Folkman (1984) states that the approach we take in a stressful situation is a process that depends on the context and two variables, the evaluations that the person does on the stress factor (how difficult is the task to be performed) and the personal, social, or cultural resources available to the person when faced with such an agent (is he/she able to perform the respective task?)^1^.

In the case of doctors, including medical students, stress is associated with multiple and major responsibilities towards the patient, time pressure, the constantly requested professional component, role conflicts, and financial problems^2^. Healthcare workers face rising pressures from increasing patient demands, complex health conditions, workforce shortages, administrative burdens, and emotional stress, intensified by evolving technologies^3^. Additionally, the recent pandemic experience influenced the medical students and made them concerned about the risk of viral infection and exposed them to mental stress as future members of the patient care teams^4^.

Medical students represent a human mass vulnerable to stress, anxiety, and depression. Students can be more emotionally vulnerable because of exam stress. Anxiety negatively affects performance and disrupts the learning process^5^. According to Carneiro^6^, 15–25% of students in medical fields face different psychiatric problems during their undergraduate training. Academic stress, caused by various factors in university and socio-familial contexts, can negatively impact students’ educational performance. Thus, the identification of risk factors, the reduction of stress, anxiety, and depression, can lead to the improvement of educational performances. Academic stress is due to the pressures from the family, teachers, as well as personal expectations. The higher the stress level, the lower the power of concentration, working memory, and consequently, school performances^7^.

The reported data in the scientific literature show different levels of stress, anxiety, and depression among dental students in different countries. More than half of U.K. dental students (56%) investigated by Collin et al.^8^ reported a high level of stress, with higher scores but without a significant statistical difference on fifth-year students compared to first-years students. Garcia et al.^9^ reported on a sample of 244 dental students in Brazil a frequency of 42.6% for stress (moderate or severe), 44.6% for anxiety (moderate or severe), and 40.2% for depression symptoms (moderate or severe), with higher self-reported levels of severe depression among students with a history of academic failure (P-value = 0.008). The frequency of moderately severe or severe anxiety (56% vs. 34.78%) and depression (49.06% vs. 37.68%) proved different between two locations in Southeastern United States (community college setting vs. university setting)^10^.

The prevalence pattern changed during COVID-19 pandemic, with a higher frequency of anxiety (moderate, severe, or severely extreme anxiety) of 56% among dental students in Malaysia^11^ and 52.2% among undergraduate dental students at the Medical University of Sofia, Bulgaria^12^. Similar prevalences of major depression (42.7%) and anxiety (44.0%) were reported in China during in COVID-19 wave two, with higher prevalence among students currently in Australia than in China and other countries (difference of 11.4% for depression and 5.6% for anxiety)^13^. Students from the different faculties of dentistry in Turkey self-reported in the first year of COVID-19 pandemic similar percentages of anxiety (~ 48%) and depression (~ 44%), and a v of stress around 37%^14^. Senior dental students (fifth and sixth year) in Lima, Peru reported during COVID-19 waves moderate, severe, or extremely severe depression in 33%, anxiety in 31%, and stress in 29%, percentages significantly higher compared to dental professional (P-value < 0.03)^15^.

Evidence concerning the frequency of stress, anxiety, and depression, among dental students in Romania remains limited. A prospective study on 2nd years dental students conducted in 2014–2015 academic year reported a decline of the WHO-5 (WHO-Five Well-Being Index) score over semester, with higher well-being levels at the beginning of the semester and pronounced decline after three consecutive mandatory examinations^16^. Our aim was to assess the occurrence of stress, anxiety, and depression among dental medicine students in Cluj-Napoca and Oradea, two prominent university centers in Transylvania, Romania. Considering that the most common forms of mental illness in the European Union are anxiety and depression disorders, it should be noted that Bihor County, where the town of Oradea is located, ranks first in the prevalence of mental illnesses, while Cluj County, where Cluj-Napoca is located, ranks 14th, according to the National Institute of Public Health^17^.

## Methods

The eligible students were informed about the study and the participants gave their consent to take part in the study. Participation in the study was voluntary, based on informed consent, with the possibility of withdrawal at any time or the possibility of choosing not to answer specific questions, with no consequences. The Ethics Committee of the “Iuliu Hațieganu” University of Medicine and Pharmacy Cluj-Napoca approved the study (no. 277/01.11.2022). Participants in the study were required to give GDPR-type written consent for their inclusion and data processing. The researchers conducted the study following the principles outlined in the Declaration of Helsinki.

### Study design and participants

A prospective longitudinal study was conducted between 1 October 2022 and 28 February 2023 targeting the students from Faculty of Dental Medicine, "Iuliu Hațieganu" University of Medicine and Pharmacy Cluj-Napoca (IHUMP), Dental Medicine specialization, Romanian section and the Dental Medicine specialization of the Faculty of Medicine and Pharmacy, University of Oradea (UO), Romania. The number of eligible students enrolled at the time of the study was 585 at IHUMP and 513 students at UO.

Without any constraints or coercions all eligible students were invited to participate. Non participation did not reflect anyhow on students’ evaluations or on teaching process.

### Research instruments and data collection

The DASS-21 (Depression, Anxiety, and Stress Scale)^18^ was used in our study, which is a tool commonly used in research and clinical settings. DASS-21 contains a set of three self-assessment scales, built to assess negative emotional states in the sphere of depression, anxiety, and stress. The Romanian version of the questionnaire, DASS-21R, has 21 items divided equally into three scales (Anxiety, Depression, Stress) and has been previously adapted and standardized for Romanian population^15^. The DASS-21R has assessed states rather than features ^19-22^,

The researchers asked the participants to fill out a paper-based survey that contained socio-demographic data and the DASS-21 survey. The collected socio-demographic data were: sex at birth, age, level of education (high school graduate with a humanities/sciences profile, other higher education graduated), where they live (Cluj-Napoca, Oradea), place of residence (urban/rural), monthly budget (monthly income), marital status (married/single/divorced—separated/widowed, in a relationship, other situations), religion, nationality, (number of) foreign languages known, and whether they work during the holidays. In the first section of DASS-21, the students were guided to respond relative to how well the statement suited thinking about their last week. The possible answers were: (0) It did not suit me; (1) It suited me to some extent or from time to time; (2) It suited me quite a lot or quite often; (3) It suited me a lot or almost all the time. The researchers informed the students that they would need about 10 min to complete the survey.

The students’ representatives distributed paper-based questionnaires to all colleagues who joined the study. The students filled out the questionnaire and returned it to their representative, who gave the filled questionnaire to the researchers. The questionnaires were distributed on Monday and collected on Friday in the same week. Data collection took place at four points in time: (1) at the beginning of the academic year, (2) before the Christmas holiday, (3) during the winter session, and (4) after the winter session. The two-page survey included on the first page a brief presentation of the study objectives and who to contact in case of questions. Students were required to create an identification code that had nothing in common with any personal data and then use that code during the four data collection periods. Demographic data were collected only in the first data collection period.

A home-made Microsoft Excel database was designed, and one researcher translated the students’ responses to electronic form. The incomplete data was identified using the Excel features and filled in when provided in the questionnaires. A second researcher double-checked the raw data and implemented correction prior to statistical analysis.

### Statistical analysis

Statistical analysis was conducted whenever all four-points data were available. The analysis was stratified on different subgroups, such as university center (Cluj-Napoca vs. Oradea), year of study (three classes: freshmen that included first year students, sophomore that included intermediate years of study, and senior that included the students in the final year of training), sex, rural vs. urban living place, previous university degree, and bachelor’s degree in humanities vs. sciences. Quantitative raw data (age, stress, anxiety, and depression scores) were tested to determine whether they followed the normal distribution within each group. The results were reported as mean (standard deviation) in case of normal distribution, respectively median and [Q1 to Q3], where Q is the quartile, when the raw data did not follow the normal distribution. Comparisons between groups (tests for independent groups) and within groups (tests for dependent groups) were performed with parametric tests for normally distributed data or non-parametric otherwise. Qualitative data were reported as absolute and relative frequencies and comparisons between groups were made using tests from the Chi-square family (Chi-square, corrected Chi-square, Fischer’s exact test), the Z test for proportions (for comparisons between two independent groups) or McNemar (for dependent groups, e.g. the presence/absence of stress, anxiety or depression between two examinations). No interim analysis was performed.

The sample size was not calculated before the study because no data is available on the Romanian population regarding stress, anxiety, and depression among the dental students. A posteriori, the power of the study was calculated, considering a level of significance equal with 5%.

The statistical analysis was done using Statistica (v.13.5, TIBCO Software, CA, USA). The significance level of 5% was applied for comparisons of the two groups. The level of significance was adjusted using the Bonferroni correction whenever over two groups were compared.

## Results

### Characteristics of the participants

The overall participation, defined as filling all four-point assessments, was 81.4%, 514/585 (87.9%) in Cluj-Napoca and 380/513 (74.1%) in Oradea. Participants from the two universities were similar in terms of year of study class, monthly budget, frequency of students in a relationship, and bachelor’s degree (Table 1).

**Table 1:** Demographic characteristics of participants by university center.

Characteristic	All (n = 894)	Cluj-Napoca (n = 514)	Oradea (n = 380)	Stat. (p-value)
Age^a^	22 [21 to 24]	22 [20 to 23]	22 [21 to 24]	-4.3 (< 0.0001)
Female^b^	701 (78.4)	416 (80.9)	285 (75.0)	4.5 (0.033)
Rural^b^	157 (17.6)	77 (15.0)	80 (21.1)	5.6 (0.0183)
Bachelor’s degree-humanities^b^	95 (10.6)	46 (8.9)	49 (12.9)	3.6 (0.0585)
Romanian nationality^b^	858 (96.0)	507 (98.6)	351 (92.4)	22.2 (< 0.0001)
Orthodox religion^b^	700 (78.3)	415 (80.7)	285 (75.0)	4.2 (0.0396)
In a relation^b^	375 (41.9)	203 (39.5)	172 (45.3)	3.1 (0.0804)
Year of study class^b^ freshman sophomore senior	164 (18.3) 567 (63.4) 163 (18.2)	97 (18.9) 317 (61.7) 100 (19.5)	67 (17.6) 250 (65.8) 63 (16.6)	1.8 (0.4152)
Monthly budget^a^	1859 [1213 to 2500]	1859 [1200 to 2500]	1917 [1300 to 2500]	-0.9 (0.3471)

^a^median [Q1 to Q3], where Q is the quartile; comparisons made with Mann–Whitney test; ^b^number (%); comparisons made with Chi-squared test; *Stat* statistics of the test; p-value = test significance

### Depression, anxiety, and stress by university center

**Fig. 1:**
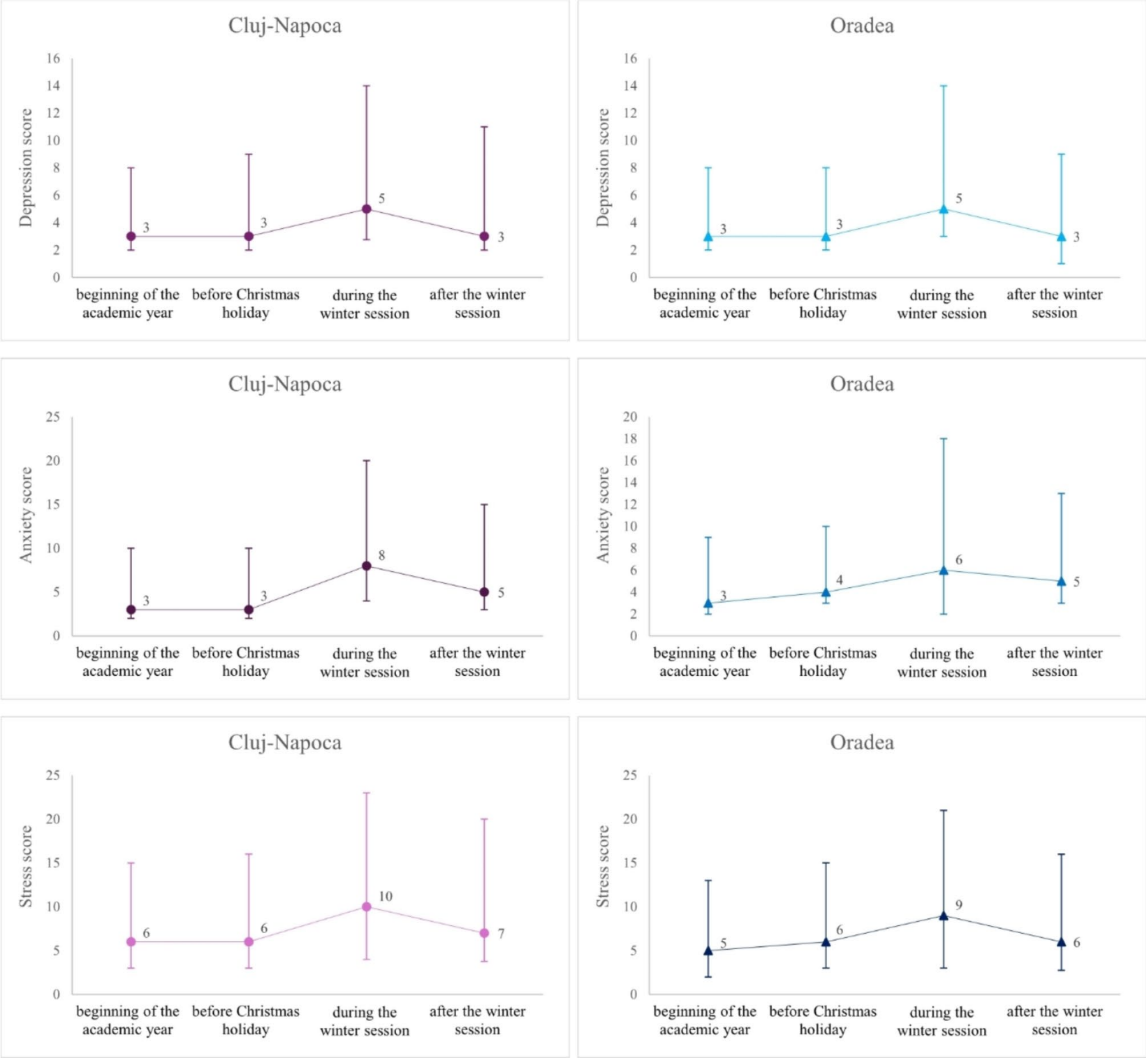
Distributions of DASS-21 scores by university center. The point within line represents the values of median, the lower whisker is given by the value of the first quartile and the upper whisker by the value of the third quartile.

No differences were observed in depression, anxiety, and stress scores between the university centers at the beginning of the university year and before the Christmas holiday. Anxiety was significantly higher during the winter session in Cluj-Napoca university center and the level of stress proved significantly higher in Cluj-Napoca dental medicine students as compared to Oradea dental medicine students (Fig. 1 and Table 2).

**Table 2:** Summary of scores by university center.

Group	Score	Beginning of the academic year (1)	Before Christmas holiday (2)	During the winter session (3)	After winter session (4)	P-Value Friedman test
All	Depression	3 [1 to 5]	3 [1 to 6]	5 [2 to 9]	3 [1 to 7]	< 0.0001
	Anxiety	3 [1 to 6.8]	3 [1 to 6.8]	7 [3 to 12]	5 [2 to 9]	< 0.0001
	Stress	5 [3 to 9]	6 [3 to 9]	10 [6 to 13]	7 [3 to 11.8]	< 0.0001
Cluj-Napoca	Depression	3 [1 to 5]	3 [1 to 6]	5 [2.3 to 9]^a,b^	3 [1 to 8]^a, b, c^	< 0.0001
	Anxiety	3 [1 to 7]	3 [1 to 7]	8 [4 to 12]^a,b^	5 [2 to 10]^a, b, c^	< 0.0001
	Stress	6 [3 to 9]	6 [3 to 10]	10 [6 to 13]^a,b^	7 [3.3 to 13]^a, b, c^	< 0.0001
Oradea	Depression	3 [1 to 5]	3 [1 to 5]	5 [2 to 9]^a,b^	3 [2 to 6]^a1, c^	< 0.0001
	Anxiety	3 [1 to 6]	4 [1.5 to 6]	6 [3 to 12]^a,b^	5 [2 to 8]^a, b, c^	< 0.0001
	Stress	5 [3 to 8]	6 [3 to 9]	9 [6 to 12]^a,b^	6 [3 to 10]^a2, b1, c^	< 0.0001
Mann–Whitney test P-value	Depression	0.6366	0.8597	0.3184	0.1628	
	Anxiety	0.1456	0.5825	0.0316	0.0307	
	Stress	0.2490	0.6267	0.1935	0.0180	

Data are summarized as median [Q1 to Q3] where Q1 is the value of the 25th percentile and Q3 is the value of 75th percentile; Mann–Whitney test P-value gave the significance of comparison between Cluj-Napoca and Oradea for each score. Friedman’s post-hoc analysis (adjusted α, p-values less than 0.0083 were considered statistically significant): ^a^P-value < 0.001 for comparison with 1; ^b^P-value < 0.001 for comparison with 2; ^c^P-value < 0.001 for comparison with 3; ^a1^P-value = 0.030; ^b1^P-value = 0.030; a2 P-value = 0.0004; ^b1^ P-value = 0.0088.

Depression scores ranged from absence (normal scores) to moderate (after the winter session) or severe (Table 3), with significantly more students with abnormal scores studying in Cluj-Napoca (17%) than in Oradea (11%) after the winter session (Chi-squared test: P-value = 0.0146).

**Table 3:** Depression, anxiety, and stress classes by university center.

		Cluj-Napoca				Oradea			
		Beginning of the academic year	Before Christmas holiday	During the winter session	After the winter session	Beginning of the academic year	Before Christmas holiday	During the winter session	After the winter session
Depression	normal	473 (92)	455 (88.5)	389 (75.7)	429 (83.5)	348 (91.6)	331 (87.1)	303 (79.7)	339 (89.2)
	mild	24 (4.7)	44 (8.6)	93 (18.1)	48 (9.3)	19 (5)	43 (11.3)	49 (12.9)	26 (6.8)
	moderate	16 (3.1)	14 (2.7)	31 (6)	37 (7.2)	8 (2.1)	6 (1.6)	28 (7.4)	15 (3.9)
	severe	1 (0.2)	1 (0.2)	1 (0.2)	0 (0)	5 (1.3)	0 (0)	0 (0)	0 (0)
Anxiety	normal	414 (80.5)	414 (80.5)	256 (49.8)	324 (63)	308 (81.1)	315 (82.9)	215 (56.6)	273 (71.8)
	mild	40 (7.8)	36 (7)	60 (11.7)	46 (8.9)	28 (7.4)	21 (5.5)	42 (11.1)	39 (10.3)
	moderate	49 (9.5)	50 (9.7)	134 (26.1)	95 (18.5)	32 (8.4)	38 (10)	72 (18.9)	53 (13.9)
	severe	8 (1.6)	13 (2.5)	62 (12.1)	41 (8)	7 (1.8)	6 (1.6)	46 (12.1)	15 (3.9)
	extremely severe	3 (0.6)	1 (0.2)	2 (0.4)	8 (1.6)	5 (1.3)	0 (0)	5 (1.3)	0 (0)
Stress	normal	479 (93.2)	484 (94.2)	431 (83.9)	440 (85.6)	366 (96.3)	367 (96.6)	318 (83.7)	336 (88.4)
	mild	28 (5.4)	25 (4.9)	63 (12.3)	58 (11.3)	12 (3.2)	10 (2.6)	54 (14.2)	39 (10.3)
	moderate	7 (1.4)	5 (1)	20 (3.9)	16 (3.1)	2 (0.5)	3 (0.8)	8 (2.1)	5 (1.3)
Data are summarized per university center as number (percentage)									

Anxiety was significantly more present in students who studied in Cluj-Napoca during the winter session (50.2% vs. 43.4%, P-value = 0.0449) and after the winter session (37.0% vs. 28.2%, P-value = 0.0057) (Table 3).

At the beginning of the academic year, significantly more students who study in Cluj-Napoca had a class of stress mild or moderate as compared to those who studied in Oradea (6.8% vs. 3.7%; P-value = 0.0424, Table 3).

### Depression, anxiety, and stress of freshmen, sophomore, and senior

**Fig. 2:**
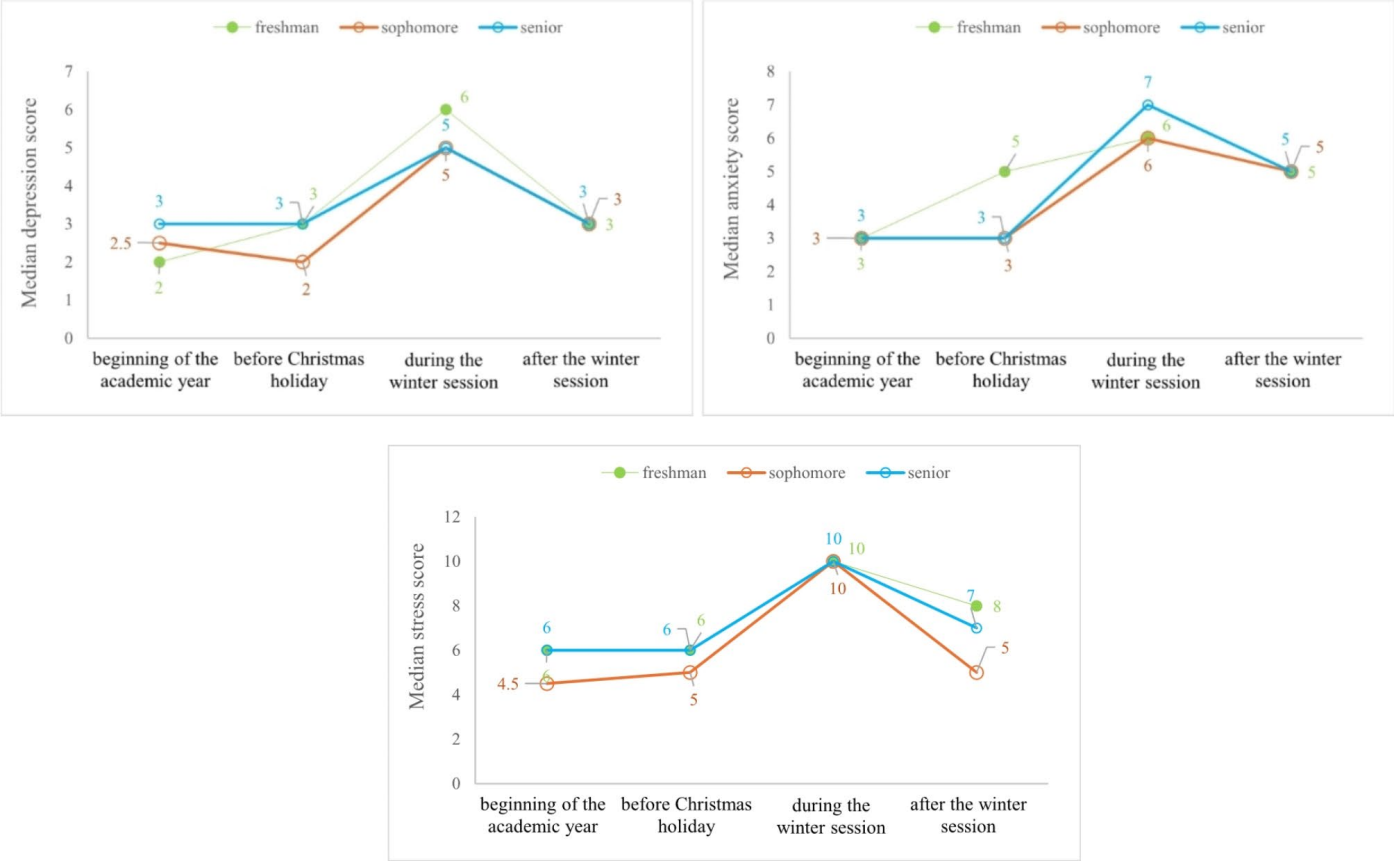
Distributions of DASS-21 median scores by academic year class (Friedman’s test p-values: senior 0.2387-depression, 0.00001-anxiety, < 0.0001-stress; sophomores: < 0.0001-depression, < 0.0001-anxiety, < 0.0001-stress; freshman: < 0.0001-depression, < 0.0001-anxiety, < 0.0001-stress).

Stress and anxiety are higher among students in the first year of the study, but the scores follow a similar trend regardless of the academic year class, with higher scores during the winter session (Fig. 2). The P-values resulted from post-hoc analysis for identified statistical significant differences are reported in Table 4.

**Table 4:** Kruskal–Wallis’s test and post-hoc analysis of students in different classes according to the year of study.

	When?	All	Freshman vs. Sophomore	Freshman vs. Senior	Sophomore vs. Senior
Depression	beginning of the academic year	0.1475	na	na	na
	before Christmas holiday	0.7582	na	na	na
	during the winter session	0.0001	ns	0.0019	0.0002
	after the winter session	0.0048	0.0054	ns	ns
Anxiety	beginning of the academic year	0.0537	na	na	na
	before Christmas holiday	0.0882	na	na	na
	during the winter session	< 0.0001	ns	0.00004	0.00002
	after the winter session	0.0050	0.0040	ns	ns
Stress	beginning of the academic year	0.0107	ns	ns	0.0244
	before Christmas holiday	0.7385	na	na	na
	during the winter session	< 0.0001	ns	0.00006	0.00002
	after the winter session	0.0083	0.0198	0.0134	ns

*na* not applicable (no significant differences between all groups);* ns*  not statistically significant.

### Depression, anxiety, and stress scores by other subgroups

**Fig. 3:**
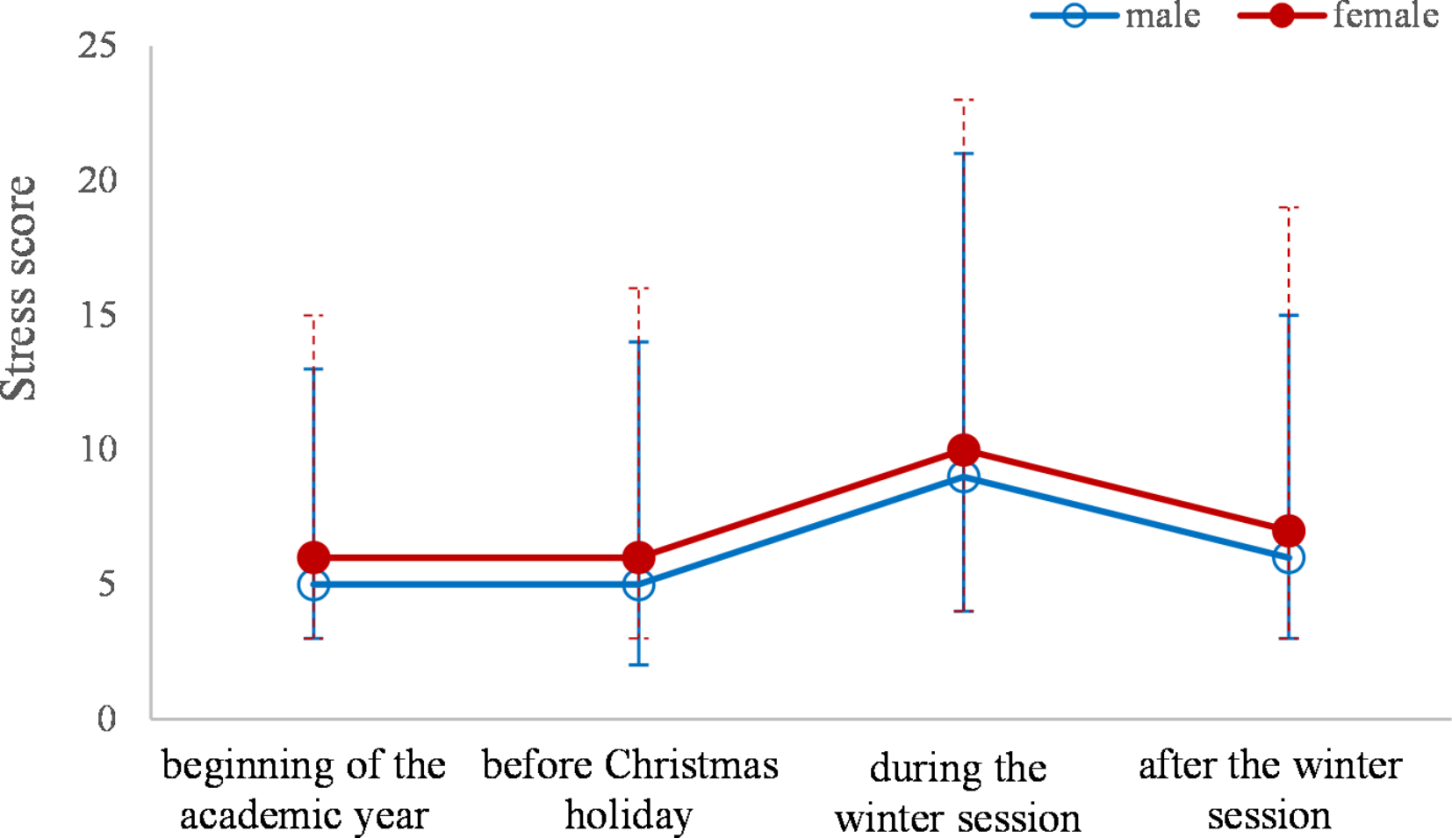
Distributions of DASS-21 stress scores by sex. The points represent the values of medians, the lower line the value of the first quartile and the upper line the value of the third quartile.

The scores of stress (Fig. 3) were significantly different between female and male only at the beginning of the academic year (median = 6 [3 to 9] for female and 5 [2 to 8], P-value = 0.0113) and after the winter session (median = 7 [4 to 12] for female and 6 [3 to 9], P-value = 0.0113), without statistically significant differences on depression and anxiety scores between female and male (P-values > 0.07).

**Fig. 4:**
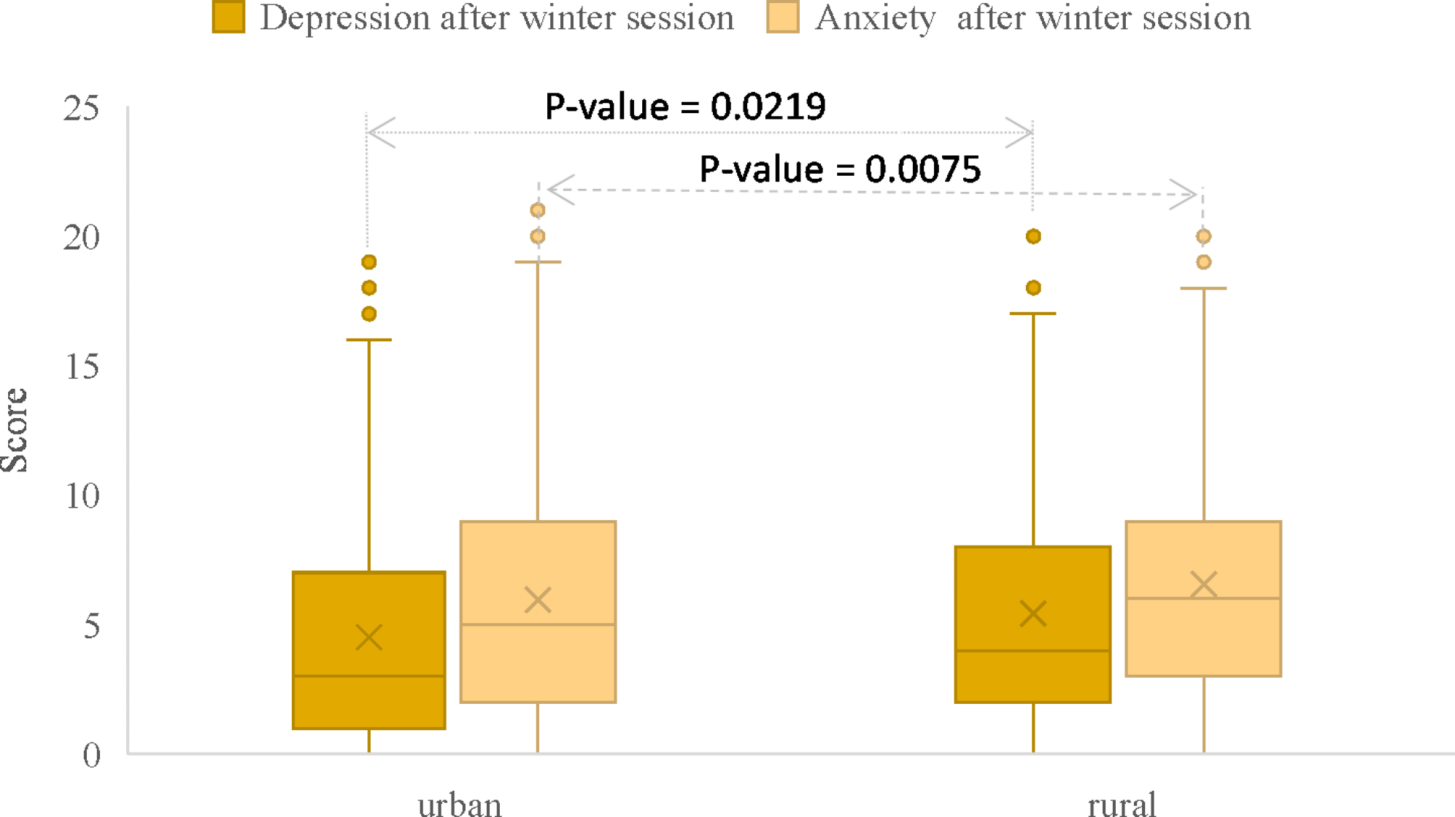
Distribution of depression and anxiety scores after the winter session for students who grew up in rural areas compared to those who grew up in urban settings.

Students who grew up in rural environments had statistically significant different scores for depression and anxiety after the winter session (rural vs. urban): 4 [2 to 8], n = 157 vs. 3 [1 to 7], n = 737, P-value = 0.0219 for depression and 6 [3 to 9], n = 157 vs. 5 [2 to 9], n = 737, P-value = 0.0075 for anxiety (Fig. 4).

The stress score at the beginning of the academic year was statistically significant different (P-value = 0.0313) among students with no previous university graduations (6 [3 to 9], n = 853) compared to students who already graduated from a university (4 [2.25 to 6], n = 42).

Students with a bachelor’s degree in humanities showed statistically significantly different anxiety (3 [2 to 7], n = 95) and stress (5 [3 to 9], n = 95) after the winter session compared to those with a bachelor’s degree in sciences (5 [2 to 9], n = 798 for anxiety and 7 [4 to 12], n = 798 for stress) (P-value = 0.0009 for anxiety and 0.0136 for stress).

No significant differences were observed in scores of depression, anxiety, or stress at any evaluated point when the students who work during summer holiday were compared to those who did not (P-values > 0.17), between those who know one foreign language compared to those who know more than one foreign language (P-values > 0.15), or between those with low monthly income as than those with more than the minimum income per month in Romania (P-values > 0.25).

## Discussion

Our results show that dental students in north-west Romanian universities experience high levels of psychological distress. Female dental students and students with no previous university graduations experience even higher levels of distress, at least in specific moments of the academic year. As far as we know, this is the first research endeavor to employ the DASS-21R questionnaire on dental students in Romania.

Dental education in Romania is a six-years program. The tuition fees are supported by the government or by the student or students’ family. To benefit from the education paid by the government, the student results must be remarkable. So, competition among students is in place, as higher the grades are, the smaller the possibility to need to pay for the education. Additionally, performances are rewarded with different scholarships. The Faculty of Dental Medicine of the "Iuliu Hațieganu" University of Medicine and Pharmacy offers six types of scholarships: scientific scholarships (180 Euro/month), four categories of merit scholarships (150, 140, 130, and 120 Euro/month), and social scholarships (116 Euro/month). At the time of the study, 151 dental students benefited from a scholarship. The Faculty of Medicine and Pharmacy in Oradea, Dental Medicine specialty, offers students four types of scholarships: merit (140 Euro/month), performance (200 Euro/month), special (200 Euro/month), and social (116 Euro/month). At the time of the study, 167 dental students benefited from the scholarship.

According to Schmitter et al., dental education is more stressful than medical education^23^. Also, according to literature, the students enrolled in dental schools in different parts of the world reported higher levels of depression, anxiety, and interpersonal sensitivity than the same age students in other fields of study ^24,25^–^28,29^.

Our study found no significant differences between students in Cluj-Napoca and Oradea during the beginning of the academic year, before the Christmas holiday, and during the winter session. However, after the winter session, a larger number of students in Cluj-Napoca compared with students in Oradea experienced mild to moderate levels of depression (17% vs. 11%, Table 3). The students who studied in Cluj-Napoca reported similar levels of anxiety during the beginning of the academic year and before the Christmas holiday compared to the students who studied in Oradea, most students reporting normal levels (Table 3). Our findings revealed that students in Cluj-Napoca had higher levels of anxiety compared to students in Oradea during the winter session (50.2% vs. 43.4%, P-value = 0.0449) and after the winter session, with levels of anxiety ranging from mild to extremely severe (37.0% vs. 28.2%, P-value = 0.0057). Stress levels did not differ significantly between universities after the Christmas holiday, and during the winter session and following the winter session. Nevertheless, at the beginning of the academic year, a significantly higher proportion of students in Cluj-Napoca (6.8%) reported mild to moderate stress levels compared to students in Oradea (3.7%) (Table 4).

The prevalence of anxiety and stress was higher during the winter session among dental students in Cluj-Napoca university center, compared with those in Oradea (Table 3). Whenever we talk about performance-related anxiety, it should be noted that the level of expectancies (both personal and interpersonal – teachers, relatives, peers) and academic standards play an important role in how we manage pressure – deadlines, the workload and how people perceive themselves managing these tasks^30^. High psychological vulnerability in anxious students is often associated with risk and failure, leading to heightened frustration. Anxious students often struggle with decision making, managing assignments, and adapting to changes.

The stress, anxiety, and depression scores showed similar patterns when we compared students according to the year of study, with significant differences during the winter session when freshman and sophomores (high scores) were compared to seniors (low scores), and after the winter session when freshman group (lower scores) was compared to sophomore group (higher scores). An explanation could be the extensive volume and complexity of subjects that must be studied, requiring young students to navigate a far more challenging adaptation process that was already passed by the seniors. Additionally, the stress score proved significantly different after the winter session in the freshman group (lower scores) compared to the senior group (higher scores). The reason behind the higher DASS-21R scores observed in our study could be attributed to the senior group’s prioritization of the specialty exam over the curriculum exams. Another possible explanation could be hypochondria, which appears when medical students imagine they have the illnesses they are studying, affecting their mental health ^31^. Anxiety can arise in students who perceive a higher chance of failure and are held to high achievement standards^32,33^. Our findings may suggest that the levels of anxiety and stress tend to rise whenever the students face experiences with little prediction. The relevant number of exam exposures they faced during the whole academic program can explain the observed differences of anxiety and stress in favor of senior students. Moreover, evidence in scientific literature exists to support the idea that repeated exposure to stressful situations creates a context of predictability and can reduce the levels of anxiety associated with the same inducing situation. Over time, it becomes more manageable to anticipate specific results and make necessary adjustments^34^. It is worth mentioning that student anxiety tends to develop when they face situations that have a direct impact on their self-esteem. Female participants in our study exhibited significantly higher stress scores at the beginning of the academic year and after the winter session, while depression and anxiety scores showed no significant differences. The explanation for this observation lies in the psychological disparities between men and women, with women having a higher tendency to express their emotions. Men tend to avoid or deny problems and, as a result, are less likely to describe themselves as stressed. Women, on the other hand, are more inclined to share how they feel, even if it does not resolve their issues. Additionally, unlike men, women display a greater vulnerability to psychologically stressful and frustrating situations. This vulnerability may stem from differences in personal resources. Moreover, in certain socio-cultural contexts, women’s lives are more stressful than men’s, as family responsibilities are added to their professional duties^35^. It is well known that women are more prone than men to express their symptoms, there also may be a commonly shared opinion that men have to endure and resist differently to life stressful situations. Social and psychological influences may also mediate this effect^36^.

Students who grew up in rural environments had higher scores associated with depression and anxiety compared to those who grew up in urban environment. Depression is mainly characterized by a sad and hopeless mindset, loss of interest in previously enjoyable activities, self-deprecating thoughts, feelings of worthlessness, low self-esteem, and reduced motivation to engage in activities. One in three young people from rural areas fears that their family will not have the financial resources needed to support them through university, and 14% are afraid they would not find accommodation in the university campus, as apartment rent are too high relative to their family incomes, according to a survey conducted by the World Vision Romania foundation^37^. Additionally, according to the same survey, 78% of young people are concerned about the rising prices^37^. In this context, losing the paid educational place in the university could contribute to the stress level. It is important to acknowledge that being in an urban academic setting exposes individuals to various situations, such as public performance evaluations and the opportunity to compare academic achievements with high achievers. This environment can foster a drive for excellence and set the standard for individuals. Additionally, the urban academic setting can serve as a place to manage emotions after failure, offering a range of resources that may lack, limited, or inexistent in rural settings^38^. Due to limited resources, students who come from rural settings may be less equipped with effective learning strategies, but also with less experience in competitive academic settings, which can predispose to frustration, sadness, and devaluation^39^.

The same mediating role of predictably may also be responsible for the levels of stress which were significantly higher among students with no previous university graduations (6 [3 to 9], n = 853) compared to students who already graduated a university (4 [2.25 to 6], n = 42). However, it should be noted that the government funds only one cycle of university studies, which increases the stress level for those who are graduates. In many cases, the difficulties encountered in finding a job drive individuals to choose a faculty that offers more opportunities or financial advantages, rather than selecting a field of interest or passion.

Our findings show that stress, anxiety, and depression are high among dental students, pointing out that interventions or preventive strategies are needed. One strategy may relate to the curriculum itself. Evidence reported by Jowkar et al. suggests that the academic factors and clinical education contribute significantly to student stress^40^. Potential modifications of the curriculum, such as adjustment of assessment methods, or integration of wellness modules into the curriculum, can create a more supportive learning environment^41^. Promoting a healthy lifestyle among dental students, such as regular physical activity, proper nutrition, and adequate sleep, can enhance students’ resilience to stress^42^. Universities can facilitate a healthy lifestyle by providing resources such as fitness programs, nutritional counseling, and workshops on time management and self-care practices. Another effective strategy for managing stress among dental students could be the implementation of structured stress management programs, such as relaxation techniques, mindfulness training, and coping mechanisms tailored to the specific challenges faced by dental students^43,44^. Another potential intervention is the existence of a peer support system within dental schools, a safe environment with individuals who understand their experiences^45^. Mental health awareness, such as regular mental health screenings, workshops on stress management, and creating accessible mental health resources, could also be a valid strategy^46,47^. Considering the findings of the present study and taking into consideration that the stress in dental education cannot be totally avoided, stress management strategies should be recommended as an integral part of the curriculum. Interaction between dental faculties and trained educational psychologists is essential for teachers to learn the latest educational methods, reducing stress and helping students cope.

Although the present study is based on a simple and concise test tool, it still has some limitations that need to be highlighted. A significant drawback is that the assessment tool relies on self-reported information, potentially leading to bias. In self-reporting, students might under-report symptoms because of recall bias (not remembering accurately), social desirability bias (answers they believe are more socially acceptable or desirable), self-perception bias (inaccurate perception of themselves), misinterpretation of questions, mood and emotional state (negative emotions could induce pessimistic responses), survey fatigue (as the same questions were asked four times the participants could rushed responses) etc. To better capture the reality, ideally, self-reporting can be duplicated by with objective measures (e.g., medical records, observational data, interviews, teaching staff assessment, etc.), anonymizing the surveys but assuring the matching for the same participant, pretesting, reducing the number of surveys per academic year, inclusion of question to evaluate the mood or similar. Furthermore, the self-reported nature of our study does not allow the assessment of psychological changes over time. The absence of more objective measures of performance, like test scores, grades, or teachers’ ratings of performance, is another limitation of our study. Study grades and other objective measurements of student performance should be used to correctly reflect the targeted relationships. Not considering factors that can influence individuals’ mental health, like sleep deprivation, lack of proper nutrition, family issues, financial problems, etc., also represents limitations. The relationship between the scholarship amount and mental health outcomes was not evaluated in our cohort, because the participants’ confidentiality could not be respected if we would ask the participants to declare personal scholarship, if any. The scholarship amount was indirectly included in the total monthly income declared by the participants, but the information regarding the scholarship could not be extracted from this data since different incomes were declared at different points of data collection. The aim of our study limited the evaluation of only two universities and only the students from one specialty, so the results reflect the participants’ experience. Thus, the reported frequencies might not reflect all dental students in Romania but could be representative for the north-west of Romania. To enhance the outcomes, future research should incorporate all dental schools in Romania and extend the time-frame to capture the association between clinical training and stress, anxiety, and depression. It also would be of interest to compare self-evaluated depression, anxiety, and stress of the targeted group with students from other disciplines and to the general population to show if the observed frequency can or cannot be attributed to the specialty.

Some students participating in our study also received medical training during COVID pandemic. School and university closures due to the COVID-19 pandemic disrupted daily routines, teaching approaches, and other aspects of everyday life. These changes deeply affected university students and young adults, with significant consequences for their mental health^48^.

High rates of emotional disturbances, including depression, anxiety, and stress, are present among undergraduate science students, indicating a need for early intervention. To promote a healthy lifestyle, students should be encouraged to dedicate sufficient time to their social and personal lives and adopt health-promoting coping strategies that can help manage stress throughout their medical studies. Academically, a well-equipped student counseling center with qualified staff should be established on campus to provide a resource for mental health support. Additionally, preventive programs should start early in medical education, addressing a range of issues from academic pressures to interpersonal and financial concerns. Identifying and addressing early signs of depressive symptoms is essential, as timely intervention can support students in managing stress, facilitating a smooth transition through their medical education and adaptation to varied learning environments. Nowadays, universities should take into consideration methods and actions, including artificial intelligence, to improve the mental health status of the students. This marks an evolution and a revolution in mental well-being^49^. Besides this, the government can play a significant role in reducing depression, anxiety, and stress among dental students by implementing policies and providing resources that promote mental health and support students’ overall well-being, supporting regular screening programs, offering more scholarships, grants, and low-interest student loans, organizing mental health awareness campaigns and providing grants for research into mental health challenges specific to dental students.

## Conclusions

The levels of depression, anxiety, and stress in evaluated undergraduate dental students are relatively high. The freshmen reported higher levels of anxiety and stress than the rest of the students, which means that, by time, they get used and accommodate with the academic environment and requirements. Moreover, the students in Cluj-Napoca reported an increase in the frequency of anxiety and stress compared with the students in Oradea all over the academic year. Students with abnormal depression and anxiety scores should receive special attention, as they require a clinical diagnosis and specialized treatment. Female students should be given more attention. For an early identification and, if needed, specialized intervention for psychological conditions, education regarding depression, anxiety, and stress should be implemented from the very beginning of studies. Moreover, strategies for stress prevention and management of these conditions should be implemented in dental schools, under the coordination of the universities and the government.

## Data Availability

The raw data used and analyzed during the current study are available from the corresponding author upon reasonable request.
